# Targeting Microglial Population Dynamics in Alzheimer’s Disease: Are We Ready for a Potential Impact on Immune Function?

**DOI:** 10.3389/fncel.2020.00149

**Published:** 2020-06-05

**Authors:** Maria Martin-Estebane, Diego Gomez-Nicola

**Affiliations:** School of Biological Sciences, University of Southampton, Southampton, United Kingdom

**Keywords:** microglia, neuroinflammation, neurodegenerative diseases, Alzheimer’s disease, proliferation

## Abstract

Alzheimer’s disease (AD) is the most common form of dementia, affecting two-thirds of people with dementia in the world. To date, no disease-modifying treatments are available to stop or delay the progression of AD. This chronic neurodegenerative disease is dominated by a strong innate immune response, whereby microglia plays a central role as the main resident macrophage of the brain. Recent genome-wide association studies (GWASs) have identified single-nucleotide polymorphisms (SNPs) located in microglial genes and associated with a delayed onset of AD, highlighting the important role of these cells on the onset and/or progression of the disease. These findings have increased the interest in targeting microglia-associated neuroinflammation as a potentially disease-modifying therapeutic approach for AD. In this review we provide an overview on the contribution of microglia to the pathophysiology of AD, focusing on the main regulatory pathways controlling microglial population dynamics during the neuroinflammatory response, such as the colony-stimulating factor 1 receptor (CSF1R), its ligands (the colony-stimulating factor 1 and interleukin 34) and the transcription factor PU.1. We also discuss the current therapeutic strategies targeting proliferation to modulate microglia-associated neuroinflammation and their potential impact on peripheral immune cell populations in the short and long-term. Understanding the effects of immunomodulatory approaches on microglia and other immune cell types might be critical for developing specific, effective, and safe therapies for neurodegenerative diseases.

## Introduction

Alzheimer’s disease (AD) is a chronic neurodegenerative disease and the most common form of dementia in the world, contributing to 60–70% of cases. It is estimated that currently over 50 million people are affected by dementia worldwide, according to the World Health Organisation and the recent report published by Alzheimer’s Disease International (ADI; Alzheimer’s Disease International, 2019). The total number of people with dementia is predicted to reach 82 million by 2030 and 152 million by 2050, causing an estimated economic burden of two trillion US$ globally (Alzheimer’s Disease International, 2019). AD is mostly diagnosed in people over 65 years-old, termed as late-onset AD (LOAD), with around 5% of AD cases being diagnosed in individuals under the age of 65, classified as early-onset AD (EOAD; Mendez, 2012). Despite these alarming figures, no disease-modifying treatment is currently available and the cause of sporadic AD is still unclear.

Clinically, AD manifests as a gradual decline in cognitive functions including loss of memory, dyspraxia, disorientation, and aphasia, accompanied by behavioral changes such as irritability, aggressiveness, anxiety, and social withdrawal (Atri, 2019). Patients are usually diagnosed based on cognitive assessments, assuming that AD neuropathologic changes will be found post-mortem. However, from 10% to 30% of patients clinically diagnosed as AD do not show AD neuropathological changes at autopsy (Jack et al., 2018), suggesting that cognitive symptoms are not the ideal method to diagnose AD. According to the updated National Institute of Aging and Alzheimer’s Association Research Framework, AD should be diagnosed by the detection of biomarkers indicative of neuropathologic changes, independently of clinical symptoms (Jack et al., 2018). This characterization is possible using PET imaging and/or assessment of biomarkers present in cerebrospinal fluid (Jack et al., 2018), although these methods are not currently being used broadly for individuals with symptoms, instead of limited to early-onset, rapidly progressive or atypical cases (Atri, 2019). The main features of the pathology of AD are the accumulation of extracellular amyloid-β (Aβ) plaques and intracellular neurofibrillary tangles of hyperphosphorylated Tau, dystrophic neurites, neuronal loss and brain atrophy (Gjoneska et al., 2015). In the last decades, several hypotheses have been explored to explain the pathogenesis of AD, being the amyloid cascade hypothesis the prevailing mechanistic theory so far. This hypothesis postulates that the neurodegeneration in AD is caused by an abnormal accumulation of Aβ protein plaques in several regions of the brain, such as the pre-frontal cortex, temporal and parietal lobe, and hippocampus, causing memory and cognitive impairment and eventually leading to dementia (Hardy and Higgins, 1992; Karran et al., 2011). Many drugs targeting this pathway have been developed and entered clinical trials in recent years. However, none of these therapies have yet been successful in preventing the development or progression of the disease. This is possibly due to the existence of alternative pathways that are disrupted in AD and not directly considered in the amyloid cascade hypothesis, which present a high therapeutic potential as alternatives or in combination with the current strategies.

Neuroinflammation associated with AD was long considered a consequence of the pathology. However, it is now well accepted that neuroinflammation is a key player in several neurodegenerative diseases, including AD. The neuroinflammatory process that takes place in these diseases is characterized by strong activation of the innate immune system, in which microglia plays a central role as the main resident macrophages in the brain (Simon et al., 2019). Microglia can respond to harmful stimuli in the brain including Aβ proteins, acting as the main regulators of the neuroinflammatory response associated with brain disease (Gomez-Nicola and Perry, 2015). In response to damage, microglia shows an activated phenotype accompanied by an increase in their proliferation and increased expression of inflammatory markers (Olmos-Alonso et al., 2016). This activation process is critical and postulated to play a beneficial role in the acute neuroinflammatory response, resulting in the engulfment of debris and dead cells to minimize and repair the brain damage (Cai et al., 2014; Calsolaro and Edison, 2016). However, the sustained activation of microglia observed in neurodegenerative diseases leads to a chronic neuroinflammatory response and an overproduction of inflammatory mediators, such as pro-inflammatory cytokines and reactive oxygen species, which are known to cause damage and neurodegeneration (Cai et al., 2014; Lyman et al., 2014; Calsolaro and Edison, 2016). The generated damage keeps microglia in an over-activated state, thus preventing these cells from returning to their homeostatic and beneficial functions and worsening the disease. It has been shown that TREM2 is critical in regulating the balance between the homeostatic and the disease-associated microglial states (Nichols et al., 2019), stimulating phagocytosis and suppressing cytokine production and inflammation (Guerreiro et al., 2013). Genetic studies have recently identified mutations of this receptor strongly associated with the risk of AD (Guerreiro et al., 2013; Jonsson et al., 2013), supporting the idea of a causative link between inflammatory cells and neurodegeneration. It has been suggested that non-steroidal anti-inflammatories have a protective role in the onset or progression of AD (Hoozemans et al., 2011), although most clinical trials to date have failed to show this beneficial effect. However, this idea is strongly supported by recent genome-wide association studies (GWAS), which have identified new single-nucleotide polymorphisms (SNPs) in immune-related genes associated with AD risk, such as the above-cited *Trem*2 (Efthymiou and Goate, 2017; Huang et al., 2017; Hansen et al., 2018; Verheijen and Sleegers, 2018). Most of these SNPs encode for proteins that are mainly expressed in microglia, strongly supporting a causal involvement of microglial cells in the development and progression of AD. These findings have attracted the effort of drug discovery programs aimed at targeting microglia-associated neuroinflammation as a potentially disease-modifying therapeutic approach for AD. In this review, we provide an overview of the main pathways controlling microglial activation and proliferation during the neuroinflammatory response and their contribution to the pathophysiology of AD. We also summarize the current therapeutic strategies to modulate microglial-associated neuroinflammation through targeting proliferation and highlight their potential impact on other immune cell populations in the systemic compartment.

## Regulation of Microglial Proliferation and Neuroinflammation in Health and AD

In recent years, GWAS studies have identified over 25 genetic loci associated with risk of LOAD, many of them related to neuroinflammation and mainly expressed in microglial cells, such as *ApoE, Spi1*, and *Trem2* (Corder et al., 1993; Guerreiro et al., 2013; Jonsson et al., 2013; Huang et al., 2017). These findings directly implicate microglial and immune genes as key players in the development and progression of AD (Efthymiou and Goate, 2017). The neuroinflammatory response in AD is characterized by the increased number of microglia cells showing an activated phenotype (Akiyama et al., 2000; Edison et al., 2008; Heneka et al., 2015; Olmos-Alonso et al., 2016), increased expression of pro-inflammatory cytokines and chemokines (Dickson et al., 1993; Fernández-Botrán et al., 2011) and an impairment in their phagocytic activity and Aβ clearance (Cai et al., 2014; Wendt et al., 2017).

### Targeting CSF1R in AD

The main system controlling the differentiation, maintenance, and proliferation of microglia in both healthy and pathological conditions is the colony-stimulating factor 1 receptor (CSF1R) pathway. CSF1R is encoded by the c-fms proto-oncogene (Sherr et al., 1985) and belongs to the type III tyrosine kinase family (Pixley and Stanley, 2004). This receptor is highly expressed by myeloid cells and its activation through the phosphorylation of the tyrosine residues stimulates many downstream signaling pathways (Pixley and Stanley, 2004; Stanley and Chitu, 2014; Wang and Colonna, 2014; Rojo et al., 2017). CSF1R genetic variants have been found by genetic screening in neuropathologically confirmed AD patients and these mutations are strongly associated with LOAD susceptibility (Sassi et al., 2018). Moreover, CSF1R upregulation and an increase in microglial proliferation have been found in post-mortem samples from patients with AD (Akiyama et al., 1994; Gomez-Nicola et al., 2013; Olmos-Alonso et al., 2016). Studies published by our group showed that microglial proliferation increases progressively in proximity to Aβ plaques in the APP/PS1 murine model of AD, suggesting that microglial activation and proliferation is triggered by Aβ deposition (Olmos-Alonso et al., 2016). It has also been shown that the pharmacological inhibition of the tyrosine kinase (TK) activity of CSF1R decreases microglial proliferation and impedes the degeneration of synapses, ameliorating the progression of the disease without modifying the levels of Aβ in the APP/PS1 model (Olmos-Alonso et al., 2016). Similar effects have been also shown in several experimental models of neurodegenerative disease, including prion disease (Gomez-Nicola et al., 2013) and amyotrophic lateral sclerosis (ALS; Martinez-Muriana et al., 2016). These results are also observed after the administration of a potent CSF1R inhibitor leading to partial depletion of the microglial population in the 3xTg (Dagher et al., 2015) and 5xFAD models (Spangenberg et al., 2016; Sosna et al., 2018) of AD-like pathology. Microglial depletion strategies were also tested in aged Tg2510 mice with no effect on tau pathology (Bennett et al., 2018). However, a recent study from our group has validated the inhibition of CSF1R as a disease-modifying mechanism in the P301S mouse model of tauopathy. This report demonstrates that inhibition of CSF1R reduces the expansion of the microglial population and the expression of pro-inflammatory cytokines such as IL-1β and TNFα at mRNA and protein levels (Mancuso et al., 2019). Blockade of microglial proliferation and the repolarization of these cells to a homeostatic phenotype attenuate neuronal degeneration and ameliorate tau pathology (Mancuso et al., 2019). This repolarization of the microglial inflammatory profile to a homeostatic phenotype has been also observed after the inhibition of CSF1R in the APP/PS1 model of AD (Olmos-Alonso et al., 2016) and other models of neurodegenerative diseases such as multiple sclerosis (Nissen et al., 2018) and a model of Parkinson’s disease (PD; Neal et al., 2020). Together, these studies provide evidence that reducing the number of microglia, or depleting them, have advantageous consequences, independently of the Aβ load, demonstrating that a disease-modifying approach for AD is achievable through targeting microglia alone.

Two independent ligands can activate CSF1R with high affinity, the colony-stimulating factor 1 (CSF-1; Stanley and Heard, 1977), and interleukin 34 (IL-34; Lin et al., 2008). Both ligands have been shown to promote microglial proliferation (Gomez-Nicola et al., 2013) but also show differential spatiotemporal expression patterns and have complementary biological functionality (Nandi et al., 2012; Wang et al., 2012). Mice lacking IL-34 (Il34LacZ) displayed an acute reduction of microglial cells in the brain and Langerhans cells in the skin, showing that IL-34 is crucial for the development and maintenance of these populations (Greter et al., 2012; Wang et al., 2012). However, the administration of anti-CSF-1 and anti-IL-34 antibodies during development or in postnatal ages revealed that CSF-1 is necessary for the colonization and maintenance of microglia population in the embryonic brain, whereas IL-34 is mainly required for microglial maintenance later during adult life (Easley-Neal et al., 2019). In adulthood, CSF-1 is widely expressed and produced by many different mesenchymal and epithelial cell types (Dai et al., 2002; Jones and Ricardo, 2013), whereas the expression of IL-34 is more tissue-restricted, mainly produced by keratinocytes located in the epidermis and neurons in the brain (Wang and Colonna, 2014), showing minimal overlap with the expression pattern of CSF-1 (Wei et al., 2010; Nakamichi et al., 2013). The role of IL-34 and CSF-1 in the maintenance of microglial cells during adulthood has been investigated in several studies during recent years. IL-34 was first shown to be required for the maintenance of microglia in the adult brain, whereas CSF-1 seemed to be mainly involved in replacing microglial cells after inflammation (Greter et al., 2012; Wang et al., 2012). However, two recently published reports have shown different effects on the microglia population after peripheral administration of specific anti-IL-34- and anti-CSF-1- monoclonal antibodies in adult mice. In the first one, Lin et al. (2019) conclude that IL-34 is crucial for the maintenance and differentiation of microglial cells in the gray matter of adult mice, whereas CSF-1 is a key player in maintaining macrophage homeostasis in several peripheral tissues such as colon and liver. However, Easley-Neal et al. (2019) show that the blockade of both molecules leads to the depletion of different microglia populations in the brain of adult mice. The anti-CSF-1 blocking antibody depleted the microglia located in the white matter more effectively, while the anti-IL-34 blocking antibody depleted the microglia in the gray matter more efficiently, phenocopying the regional expression pattern of each ligand (Easley-Neal et al., 2019). Taking together, all this evidence suggests that CSF-1 and IL-34 are required differentially during development and for the maintenance of the microglial population in the adult brain. In AD and AD-like transgenic mice, CSF-1 was shown to be upregulated and played an essential role in the proliferation of microglia occurring as a consequence of the pathological activation in disease (Murphy et al., 2000; Vincent et al., 2002). Regarding IL-34, Mizuno et al. (2011) showed that IL-34-treated microglia attenuates the neurotoxic effects of Aβ in neuron-microglia co-cultures by promoting microglial uptake and metabolism of Aβ. The neuroprotective role of IL-34 in this system seemed to be regulated by transforming growth factor β-1 (TGFβ-1). The inhibition of the TGFβ-1 receptor results in an increased microglial proliferation driven by IL-34 and the suppression of the observed neuroprotective effect of IL-34-treated microglia. These observations suggest that TGF-β produced by these cells acts as a negative regulator of microglial proliferation, improving the neuroprotective feature of microglia (Ma et al., 2012). In the APP/PS1 model of AD, the administration of IL-34 in the brain ameliorates the impairment of associative learning (Mizuno et al., 2011). These studies provided evidence that modulation of these cytokines may also be an approach to control the microglia population in the context of neurodegenerative diseases, as an alternative method to CSF1R modulation.

### Role of PU.1 in the Modulation of Microglial Proliferation and Activation

The transcription factor PU.1 is also an important player in the development, proliferation, and maintenance of microglia. PU.1, encoded by the gene *Spi1*, belongs to the ETS-family of transcription factors and is a master regulator of myeloid and lymphoid development and function (Scott et al., 1994; McKercher et al., 1996; Dakic et al., 2005). This transcription factor binds to a purine-rich DNA sequence (PU.1-box) located upstream of the promoter of its targets and activates the expression of a great number of downstream genes (Pham et al., 2013). PU.1 is necessary for the correct development and functional maintenance of the microglial population since it is continuously expressed from erythromyeloid progenitors to adult microglia (Kierdorf et al., 2013; Smith et al., 2013). PU.1-deficient mice show a complete loss of microglia and other myeloid cell types such as macrophages and monocytes, indicating that PU.1 regulates key genes involved in the differentiation and the maturation of hematopoietic cells and also microglia (McKercher et al., 1996; Beers et al., 2006). Satoh et al. (2014) identified 5,264 *Spi1* target protein-coding genes in the mouse microglial cell line BV2 by chromatin immunoprecipitation (ChIP)-seq analysis, including *Spi1* itself, the transcription factors *Irf8* and *Runx1*, *Aif1* (*Iba1*), *Csf1r* and its ligands *Csf-1* and *Il-34*. Interestingly, two-thirds (63%) of the genes that define the microglial sensome are PU.1 targets, suggesting that PU.1 plays a pivotal role in the regulation of specific microglial functions (Satoh et al., 2014) such as cell survival, phagocytosis, antigen presentation, and morphology. Recently, a GWAS study has identified a common haplotype, rs1057233 (G), located in a previously reported AD risk locus (CELF1), which displays a reduced expression of PU.1 in human myeloid cells associated to delayed age of onset of AD (Huang et al., 2017). The alteration of PU.1 levels in mouse and human microglial cells affected the expression of many AD risk genes (Huang et al., 2017) and their phagocytic activity (Smith et al., 2013; Huang et al., 2017; Rustenhoven et al., 2018). The activation of microglia through PU.1 is critical for the progression of Alzheimer’s disease (Gjoneska et al., 2015), emphasizing the role of microglia at the onset of the disease. Similarly, the activation of microglia through PU.1 is observed in response to mutant Huntingtin aggregates present in Huntington’s disease, hypoxic-ischemeic insults and traumatic injury-induced neurodegeneration (Walton et al., 2000; Crotti et al., 2014; Zhou et al., 2019). Moreover, a recent study published by Litvinchuk et al. demonstrated that PU.1 and the transcription factors Irf8 and Runx1 were significantly upregulated in FACS-isolated microglia in the PS19 mouse model of tauopathy and AD (Litvinchuk et al., 2018). Together, these findings suggest that changes in the expression level of PU.1 may be a shared feature underlying several neurological disorders and highlight its modulation as a potential mechanism to control neuroinflammation. Studies using PU.1^−/−^ mice have shown that complete loss of function of PU.1 results in stem cell failure (Antony-Debre et al., 2017), multiple hematopoietic abnormalities and, ultimately, developmental mortality (McKercher et al., 1996), highlighting the importance of achieving partial inhibition of PU.1 to understand its potential roles in disease. Newly described pharmacological PU.1 inhibitors have been recently developed (Munde et al., 2014; Stephens et al., 2016) and tested in murine and human acute myeloid leukemia (AML; Xeno) transplantation models, decreasing leukemia progression without affecting normal hematopoietic differentiation (Antony-Debre et al., 2017). These small molecules disrupt the interaction of PU.1 with its binding sites next to the promoters of target genes and lead to the downregulation of PU.1 transcriptional targets, holding a high potential as tool compounds for evaluating the role of PU.1 in neurodegenerative diseases.

## Current Therapeutic Strategies Targeting Microglia Population Dynamics and Potential Side Effects on Peripheral Populations

To date, drugs available for AD are restricted to relieve its symptoms, with no treatments able to stop or delay the progression of this disease. The cognitive problems in early-to-moderate AD are treated with Acetylcholinesterase inhibitors (Donepezil, Rivastigmine, and Galantamine) which block the degradation of acetylcholine and enhance cholinergic neurotransmission, deficient in AD. Additionally, patients are treated with Memantine which protects against the glutamate excitotoxicity seen in neurodegenerative disorders such as AD. Currently, there are an estimated number of 132 agents in clinical trials for the treatment of AD, 30 in phase I of development, 74 in phase II, and 28 in phase III. Among these agents, 96 (73%) are disease-modifying therapies; 38 (40%) and 17 (18%) of these have amyloid and tau as the primary target, respectively (Cummings et al., 2019). However, multiple failures to stop AD using similar strategies in the past have considerably increased the interest in other targets, such as those related to neuroinflammation, with three agents currently in phase II and two agents in phase III clinical trials (Cummings et al., 2019). Also, recent genetic evidence links microglia function to AD pathogenesis, placing the spotlight on microglia as a potential target to treat AD.

**Figure 1:**
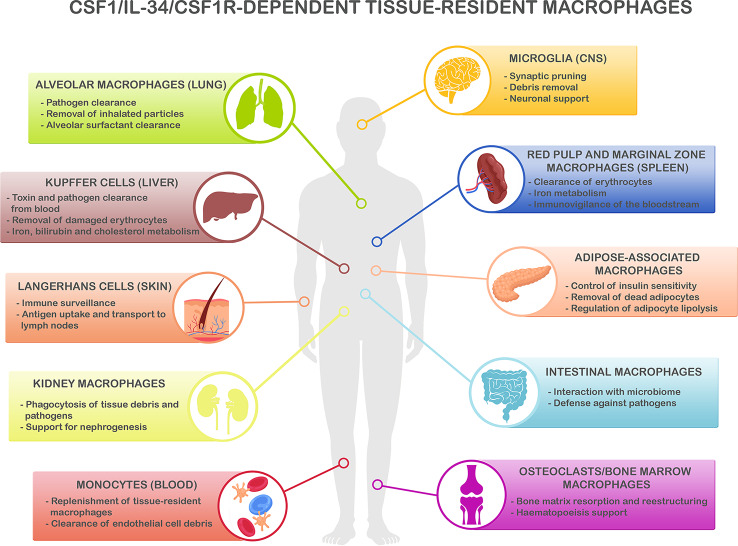
CSF1R/CSF-1/IL-34-dependent tissue-resident macrophage key functions. CSF1R-, CSF1- and IL-34-dependent macrophage populations perform key functions to maintain homeostasis in different organs. Microglia, the main resident macrophages in the brain, is responsible for many critical functions during development and adulthood including support of neurogenesis, synaptic formation, and pruning, and phagocytosis of apoptotic neurons and debris in the extracellular space (Colonna and Butovsky, 2017; Li and Barres, 2018). In the lungs, alveolar macrophages are responsible of the clearance of inhaled pathogens and particles (Maus et al., 2002; Davies et al., 2013), and they also play a critical role in the maintenance of alveolar homeostasis by clearing lipoprotein-containing alveolar surfactant produced by alveolar epithelial cells (Dranoff et al., 1994; T’Jonck et al., 2018). Kupffer cells, the resident macrophages in the liver, are involved in many immune and homeostatic functions such as clearing gut-derived toxins and pathogens from the blood, removal of damaged erythrocytes, as well as iron, bilirubin, and cholesterol metabolism (Ganz, 2012; T’Jonck et al., 2018). The spleen contains multiple subsets of macrophages such as red pulp macrophages, located in the red pulp of the organ. They play a vital role in the clearance of senescent red blood cells and iron recycling (Kurotaki et al., 2015; T’Jonck et al., 2018). Next to red pulp macrophages, the spleen also contains marginal zone macrophages that are involved in the detection of antigens present in the bloodstream (den Haan and Kraal, 2012; Kierdorf et al., 2015). Adipose-associated macrophages, present in the pancreas and adipose tissue all over the body, fulfill different functions such as removal of dead adipocytes, regulation of adipocyte lipolysis, storage and release to the bloodstream of excessive adipocyte-released lipids, and participation in the control of insulin sensitivity (Odegaard et al., 2007; Boutens and Stienstra, 2016; T’Jonck et al., 2018). Macrophages in the gastrointestinal tract continuously interact with the intestinal microbiome and maintain intestinal homeostasis regulating the immune response to commensals and defending the tissue against pathogens (Davies et al., 2013; Zigmond and Jung, 2013). Langerhans cells are resident macrophages in the skin, involved in tissue surveillance, and uptake, and transport of antigens to the skin-draining lymph nodes (Chorro and Geissmann, 2010; Kierdorf et al., 2015; T’Jonck et al., 2018). Renal macrophages play several roles such as surveillance of the environment, phagocytosis of pathogens, and debris present in the extracellular matrix as well as support for nephrogenesis (Nelson et al., 2012). Circulating Ly-6C^lo^ monocytes are the predominant macrophage subset in the blood, acting as “intravascular housekeepers” in the clearance of endothelial cell debris as well as entering other tissues for the replenishment of tissue macrophage populations (Carlin et al., 2013; Gordon et al., 2014). Finally, different types of macrophages play critical roles in the bone. Osteoclasts are large multinucleated macrophages in charge of maintaining bone homeostasis and structure by resorption of the bone matrix produced by osteoblasts (Davies et al., 2013; T’Jonck et al., 2018), whereas bone marrow macrophages support erythropoiesis and maintain hematopoietic stem cells in stem cell niches (Chow et al., 2011, 2013; Davies et al., 2013). Considering the shared myeloid lineage of all these macrophage populations, it is anticipated that the immune and homeostatic key functions above described are susceptible to be affected by the immunomodulatory strategies to reduce neuroinflammation.

Several microglial genes identified as robustly-associated with the risk of LOAD are now under investigation as potential targets for drug development, such as APOE, TREM2, CD33, and CR1, amongst others (for review see; Biber et al., 2019; Hemonnot et al., 2019). Despite the importance of the above-cited targets and their strong link with AD pathogenesis, here we focus on those related to the modulation of the dynamics of the microglial population. Microglial cells share many functions, genes, and developmental lineage with other cells of the myeloid lineage across different organs (Hoeffel and Ginhoux, 2018), which are required for the proper functioning of the immune system (Figure 1). Because these gene expression signatures are conserved, it is extremely important to evaluate the impact of anti-neuroinflammatory agents on the broader immune system. The therapeutic benefit of influencing a given cellular function in a given pathology may result in the alteration of the natural balance of the broader immune system, with unknown consequences frequently not taken into consideration. Here, we review the potential side effects of manipulating immune-related pathways on other populations of immune cells, located in different organs of the systemic compartment.

Importantly, people with dementia usually have co-morbidities ranging from two to eight health conditions (Nelis et al., 2019). It is accepted that people with dementia have an average of four co-morbidities, compared to an average of two in people without dementia of similar age (Poblador-Plou et al., 2014). A recent study across various care settings has reported that 61% of the people with AD had three or more co-morbidities (Nelis et al., 2019). Over 90% of people with dementia have at least one co-morbidity, with some of these being often undiagnosed (Browne et al., 2017). Some of the main co-morbidities significantly associated with dementia are cardiac arrhythmia, hypertension, congestive cerebrovascular disease, diabetes, and depression (Nelis et al., 2019). A common feature of several co-morbidities is a dysfunctional immune response. For example, obesity-related metabolic disorders, which are also risk factors for AD, are associated with alterations in the inflammatory status (Nguyen et al., 2014; Saltiel and Olefsky, 2017). Similarly, increasing evidence in recent years has demonstrated the important role of inflammation in the pathophysiology of diabetes (Tsalamandris et al., 2019), an age-related chronic disorder highly prevalent in AD patients (Newcombe et al., 2018; Nelis et al., 2019). Two of the most prevalent conditions associated with normal aging and dementia are cardiovascular disease and hypertension, both closely related to the above-cited metabolic disorders (Lopez-Candales et al., 2017; Nelis et al., 2019). Similar to those, recent studies have supported the causal role of chronic inflammation in the development of these cardiovascular conditions (Lopez-Candales et al., 2017; Ruparelia et al., 2017). Also, the incidence of systemic infections, such as urinary tract infection (UTI) and gum disease, is increased in Alzheimer’s, further accelerating the cognitive deterioration (Doraiswamy et al., 2002; Dominy et al., 2019). Psychiatric disorders with elevated prevalence in AD, such as depression, have also been related to peripheral and central chronic inflammation, which seem to drive changes in neurotransmitters leading to depressive symptoms (Felger, 2019). On the opposite spectrum, a growing body of evidence suggests an inverse link between the incidence rates of cancer and AD, even though both are age-related disorders with significant immune involvement (Majd et al., 2019). Taken together, this evidence highlights the fact that the co-existence of age-related comorbidities is a crucial aspect to consider in the development of immunomodulatory therapeutic strategies for treating AD, which in turn may compromise the responsiveness and immune control of these co-morbidities.

### Inhibiting CSF1R in AD: Target Validation Studies

The therapeutic potential of inhibiting CSF1R has been proposed for inflammatory diseases, autoimmune disorders, bone diseases, and cancer (Burns and Wilks, 2011). Targeted inhibition of CSF1R signaling has the potential to treat a wide variety of neurodegenerative diseases associated with chronic neuroinflammation such as AD, PD, Huntington’s disease, ALS, multiple sclerosis, and psychiatric disorders. CSF1R can be blocked by at least two different approaches: (i) using small-molecule inhibitors targeting the TK activity of the receptor or (ii) antibodies that bind the receptor and block the interaction between CSF1R and CSF-1/IL-34. The first neutralizing monoclonal antibody against CSF1R, AFS98, was produced by Sudo et al. (1995) and was shown to be effective in the control of CSF1-related functions in pathology. Some examples of its effectiveness are the reduction of macrophage accumulation in atherosclerotic lesions and diabetic nephropathy, the reduction of infiltrating macrophage proliferation in renal allografts and damaged skeletal muscle (for review see Hume and MacDonald, 2012), and the local inhibition of microglial proliferation in the prion disease model ME7 (Gomez-Nicola et al., 2013). In contrast with these results, prolonged treatment with a different monoclonal anti-CSF1R antibody, M279, selectively removed tissue macrophages, including macrophages inside the tumors, but had no protective effect in several models of inflammation (MacDonald et al., 2010). This antibody is incapable of crossing the blood-brain barrier (BBB), depleting microglia in the retina but not affecting the brain (Hume and MacDonald, 2012). It has also been shown that after prolonged treatment with M279 bone density and trabecular volume are increased due to the ablation of osteoclasts, preventing the reduction in bone mass observed in female mice with age. This long-term effect on bone remodeling suggests that M279 could potentially be used as a treatment for osteoporosis (Sauter et al., 2014). Importantly, a side effect of CSF1R blocking antibodies is related to the role of CSF1R in the clearance of CSF-1 from the circulation by endocytosis (Hume and MacDonald, 2012). CSF1R blockade causes a massive increase in the concentration of circulating CSF-1, and rebound monocytopoiesis (Hume and MacDonald, 2012). However, this effect does not occur when the TK activity of the receptor is blocked by kinase inhibitors, since this activity is not required for the internalization of CSF-1 (Hume and MacDonald, 2012). One of the most important features of kinase inhibitors, compared to antibodies, is that small molecules can block autocrine actions of endogenous CSF-1, which is highly expressed in some mouse inflammatory macrophages and drives the expression of inflammatory cytokines (Hume and MacDonald, 2012). One of the most selective and best characterized of the available TK inhibitors probably is GW2580. GW2580 inhibits the growth of CSF1-dependent tumor cells (Conway et al., 2005) and the recruitment of macrophages into growing tumours (Priceman et al., 2010). It has also been shown to exhibit antitumor activity in AML by blocking paracrine production of hepatocyte growth factor and other cytokines signaling from support cells (Edwards et al., 2019). GW2580 has beneficial effects, by blocking microglial proliferation, in several experimental models of multiple sclerosis (Crespo et al., 2011), prion disease (Gomez-Nicola et al., 2013), AD (Olmos-Alonso et al., 2016), ALS (Martinez-Muriana et al., 2016), spinal cord injury (Gerber et al., 2018) and PD (Neal et al., 2020). Using the APP/PS1 model of AD-like pathology, we found diminished synaptic degeneration and improved behavioral and performance and learning after chronic inhibition of CSF1R with GW2580 (Olmos-Alonso et al., 2016). A different CSF1R inhibitor with significant *in vivo* data available is Ki20227. This inhibitor has been shown to reduce the number of macrophages and associated pathology in models of inflammatory arthritis (Ohno et al., 2008) and encephalomyelitis (Uemura et al., 2008). However, Ki20227 reduced the numbers of Ly6G^+^ granulocytes, an effect that generates concerns about its specificity. There are some other TK inhibitors that block CSF1R but also have affinity for other kinases, as the orally available JNJ-28312141 (Hume and MacDonald, 2012). This inhibitor has specificity against CSF1R but also the related receptor FLT3 and has been shown to reduce macrophage numbers and limit tumour growth in several models of transplanted tumours as well as in a FLT3-dependent subset of AML (Manthey et al., 2009). Despite J&J had JNJ-28312141 in phase II clinical trials for the treatment of rheumatoid arthritis (RA), this was discontinued and replaced by JNJ-40346527. This CSF1R inhibitor has been recently shown to repolarise microglia to a homeostatic phenotype and attenuate tau-induced neurodegeneration resulting in functional improvement in the P301S mouse model of tauopathy (Mancuso et al., 2019). Currently, JNJ-40346527 is in phase II ongoing trials for AML (NCT03557970) and phase Ib ongoing trials for AD (NCT04121208). Recently, a novel family of inhibitors developed by Plexxicon has been described to have a potent activity over CSF1R. PLX3397 (Pexidartinib) was shown to inhibit the survival of microglia and cause a fast depletion of the population in the healthy brain (Elmore et al., 2014). PLX3397 was shown to prevent neuronal degeneration, improving cognitive functions in the 5xFAD model of AD-like pathology (Spangenberg et al., 2016; Sosna et al., 2018). Similar results were obtained using the inhibitor PLX5622 in the 3xTg AD model (Dagher et al., 2015). However, PLX3397 also causes a potent inhibition of c-kit and PDGFRβ (Patwardhan et al., 2014), which may confound the observed effects on the microglial population. The inhibition of PDGFRβ and loss of PDGFβ signaling would affect the survival of NG2 pericytes, consequently damaging the BBB and influencing neurodegeneration (Montagne et al., 2017). Despite the unknown side effects of these molecules in brain disease, PLX3397 is currently in phase II ongoing trials for several types of tumours such as sarcoma and glioblastoma (NCT01790503; NCT02584647). Another small molecule in development for AD is Masitinib, a pan-kinase TK inhibitor. AB Science SA is using Masitinib in phase III trials for patients with mild to moderate AD (NCT01872598), a wide variety of tumours such as gastrointestinal stromal tumours (NCT01694277), ALS (NCT02588677; NCT03127267) and multiple sclerosis (NCT01433497), based on the activity of the compound over CSF1R or c-kit, depending on the specific disease mechanism. In summary, many approaches have been designed to target the activity of CSF1R under neuroinflammatory conditions, and in the coming years, the field will collect valuable clinical information about their potential efficacy in AD.

### The Systemic Impact of CSF1R Inhibition: Can Selectivity and Safety be Improved?

According to the above-cited studies, blocking the expansion of the microglial population results in a significant reduction of neuronal degeneration, leading to an improvement in the disease symptoms and survival. These results provide strong evidence of the potential application of CSF1R tyrosine kinase inhibitors as a promising approach to tackle microglial proliferation in neurodegeneration. However, although many CSF1R inhibitors are progressing to clinical trials, little is known about the impact of these approaches on the innate immune system. CSF1R is expressed in many cell types of the myeloid lineage, including tissue-resident macrophages, dendritic cells, and their precursors (Chitu and Stanley, 2017). Therefore, the inhibition of CSF1R would not only affect microglia but also other tissue-resident myeloid populations, possibly causing an immunosuppressive effect.

A potential approach to block this pathway more selectively is by modulating the binding of its ligands, CSF-1 and/or IL-34, to increase tissue specificity and reduce side effects. This approach is based on the differential tissue-selectivity and functions of CSF-1 vs. IL-34, reported in the literature and discussed previously. The blockade of both ligands can be achieved by the use of specific antibodies directed against these cytokines, with beneficial effects in murine models of arthritis, colitis, and ileitis (Lin et al., 2019). However, blockade of both ligands, separately or in combination, leads to altered macrophage homeostasis in healthy mice, reducing the numbers of macrophages in the intestine, liver, kidney, skin, bone marrow and microglia in the brain (Easley-Neal et al., 2019; Lin et al., 2019). In contrast to these observations, a recent study from our group shows that monocyte and macrophage populations in peripheral tissues were not affected after the selective blockade of IL-34 in healthy mice, except for the skin-resident Langerhans cells (Obst et al., 2020). However, the number of monocytes and macrophages were significantly decreased after blockade of CSF1R, following the wider expression of the receptor. Despite the microglial population was not affected after systemic administration of anti-IL-34 antibodies, due to their low brain penetrance, we observed a local reduction of microglia proliferation after the intracerebral injection of anti-IL-34 antibodies in mice infected with prion disease, showing that IL-34 is a key driver of microglial proliferation in the context of neurodegenerative disease (Obst et al., 2020). Our results support that modulation of the microglial response *via* IL-34 blockade could be a potential and more selective therapeutic approach in neurodegenerative diseases (Obst et al., 2020). A similar therapeutic approach modulating the granulocyte-macrophage colony-stimulating factor (GM-CSF) instead of targeting its receptor is currently in phase II clinical trial for AD (NCT01409915), which has been recently completed although no results have been published yet. Testing of this recombinant human factor, named as Sargramostim, for AD is based on published results regarding GM-CSF role in AD mouse models, in which GM-CSF seems to reduce brain amyloidosis and reverse cognitive impairment by increasing microglial density and their activation state (Boyd et al., 2010; Kiyota et al., 2018). However, some studies have reported an increased expression of GM-CSF in AD patients (Tarkowski et al., 2001) and a beneficial role of blocking this factor using an anti-GM-CSF antibody in a mouse model of AD (Manczak et al., 2009). Nevertheless, the potential side effects of this approach on other myeloid populations are unknown, supporting the idea that more studies are necessary to understand the effects of modulating these molecules in neurodegenerative diseases and their potential on-target effects on tissue-resident macrophages.

The functions of CSF-1, IL-34, and CSF1R in monocyte-macrophage differentiation have been demonstrated through the study of specific genetic mutations in mice, rats, and humans (Hume and MacDonald, 2012; Chitu and Stanley, 2017). Mice and rats with *Csf-1* loss-of-function mutations have deficiencies in many tissue macrophage populations and are severely osteoporotic, due to the lack of osteoclasts (Dai et al., 2002). Pleiotropic effects including severe postnatal growth retardation, neurological and reproductive deficiencies, highlight the important trophic roles of CSF1-dependent macrophages (Wynn et al., 2013). By contrary, IL-34 mutation is less severe, only depleting microglia and Langerhans cells, consistent with its restricted regional expression (Wang et al., 2012). CSF1R knockout mice display a severe phenotype characterized by limited survival after the weaning phase (Chitu et al., 2016). Interestingly, a recent study has shown that genomic deletion of FIRE, a highly conserved *Csf1r* enhancer, ablates specifically microglia and resident macrophages in some tissues such as the skin, kidney, heart, and peritoneum (Rojo et al., 2019). They demonstrate that Csf1r^ΔFIRE/ΔFIRE^ mice are healthy and fertile, not showing the severe postnatal growth retardation and developmental abnormalities observed in Csf1r−/− rodents (Rojo et al., 2019). In humans, the hypomorphic mutation in CSF1R causes hereditary diffuse leukoencephalopathy with spheroids, a disease originated from the loss of myelin and the destruction of axons (Wynn et al., 2013). Homozygous mutations in CSF1R in human leads to premature death, linked to severe brain abnormalities including hydrocephaly, hypomyelination, and abnormal bone growth (Oosterhof et al., 2019). Given the central role of macrophages in fighting infection (Figure 1), long-term blockade of the CSF1R/CSF-1/IL-34 axes could compromise the response to infection. Mice infected with *Listeria monocytogenes* and treated with antibodies against CSF-1/IL-34 were more susceptible to the bacterial infection, showing that these approaches might be immunosuppressive in the rodent *Listeria* model (Lin et al., 2019). Similar results were obtained in a model of viral encephalitis, where the inactivation of CSF1R using a tyrosine kinase inhibitor reduced circulating antigen-presenting cells in the blood leading to a higher susceptibility to lethal West Nile virus infection (Funk and Klein, 2019). This study shows the importance of CSF1R in myeloid cell responses that involve the restriction of viral replication, and the local restimulation of recruited antiviral T cells within the CNS (Funk and Klein, 2019). On the other hand, a different CSF1R TK inhibitor showed good safety and tolerability profile after 3 months of treatment in patients with RA, causing only an alteration in Kupffer cell function (Figure 1; Genovese et al., 2015). Kupffer cells may have a role in clearing several serum enzymes, including alanine aminotransferase and aspartate aminotransferase, which are often used as indicators of hepatic injury during medical tests and clinical trials (Radi et al., 2011; Lin et al., 2019). The reduction in the population of Kupffer cells after treatment with anti-CSF-1/IL-34 antibodies correlated with an increase of these enzymes in the serum of rodents and monkeys, although no histopathological evidence of liver injury was observed (Radi et al., 2011; Lin et al., 2019). Importantly, the detection of high liver enzyme activity, unrelated to a hepatocellular injury, may compromise clinical monitoring of liver injury, an aspect to take into consideration with therapeutics that target macrophages Lin et al., 2019). Bone formation and resorption is also a process influenced by CSF1-CSF1R signaling (Figure 1). CSF-1 is produced in the bone marrow by osteoblasts, binding to CSF1R located on the surface of osteoclast precursors, giving rise to the formation of osteoclasts (El-Gamal et al., 2018). Mice lacking CSF-1 are unable to generate osteoblasts, leading to low bone density and osteoporosis (El-Gamal et al., 2018). However, CSF1R inhibition would likely lead to increased bone density and abnormal bone growth due to a decrease in osteoclast numbers. This may result in the development of Paget’s disease, which is characterized by enlarged and misshapen bones. Another effect of CSF-1 deficiency in the macrophage-deficient Csf1^op^/Csf1^op^ model is an insulin mass deficit due to the reduction of pancreatic β cell proliferation and abnormal islet morphology in the pancreas (Banaei-Bouchareb et al., 2004). The addition of CSF-1 to embryonic pancreas explants caused a higher differentiation of β cell and increased production of insulin (Geutskens et al., 2005). However, macrophage ablation in the pancreas and adipose tissue after long-term anti-CSF1R treatment (Figure 1), had no effect on average size or distribution of β cells within islets of Langerhans, detected by immunostaining for insulin (Sauter et al., 2014). Despite the decrease in tissue resident macrophages in many organs after the treatment with an anti-CSF1R antibody, Sauter et al. (2014) did not observe any overt pathology in hematoxylin and eosin sections of different organs (Sauter et al., 2014). In summary, CSF1R/CSF-1/IL-34 blocking strategies have different effects on tissue-resident macrophages and other cell types of the systemic compartment, leading to a dysregulation of the tissue homeostatic functions (Figure 1). Likewise, any therapeutic approach directed against potential microglial targets, e.g., TREM2, inflammasome, among others, is expected to have a comparable impact on peripheral immune cell populations and organ function. Therefore, we need further investigation of the potential side effects of manipulating immune-related pathways to modulate the microglial population during neuroinflammation, in order to design and develop highly specific therapeutic agents.

## Conclusion

Over recent years the field of study of the contribution of neuroinflammation to AD has undergone a revolution. The number and quality of preclinical studies have increased, leading to some very promising early clinical studies, using agents directed against neuroinflammatory targets. In the coming years, this field will finally start to collect some critical clinical data, which will allow, once and for all, to address the hypothesis that neuroinflammation is a driver of neurodegeneration in AD. These early promising studies should not distract the field from trying to find better, more refined, approaches, to overcome the anticipated significant impact over the broader immune system. In the meantime, it is crucial to start to understand the impact of targeting key neuroinflammatory pathways on the function of other tissue-resident macrophages, and the key organ functions they are responsible for. If any of the postulated anti-neuroinflammation agents succeeded to progress to longer trials or eventually to enter the market, it is anticipated that the AD target population would be exposed for very prolonged periods to agents influencing their immune balance. Considering AD patients are often multimorbid, this would have unknown consequences over their responsiveness to infection or the control of their immune-related co-morbidities.

## Author Contributions

MM-E and DG-N designed the structure, content of the manuscript and drafted the manuscript. MM-E wrote the manuscript and prepared the figures.

## Conflict of Interest

The authors declare that the research was conducted in the absence of any commercial or financial relationships that could be construed as a potential conflict of interest.

## Abbreviations

AD: Alzheimer’s disease
GWASs: genome-wide association studies
Aβ: amyloid-β
LOAD: late-onset AD
EOAD: early-onset AD
SNPs: single-nucleotide polymorphisms
CSF1R: colony-stimulating factor 1 receptor
ALS: amyotrophic lateral sclerosis
CSF-1: colony-stimulating factor 1
IL-34: interleukin 34
TGFβ-1: transforming growth factor β-1
AML: acute myeloid leukemia
BBB: blood-brain barrier
PD: Parkinson’s disease
RA: rheumatoid arthritis
TK: tyrosine kinase.
